# Social values and the corruption argument against financial incentives for healthy behaviour

**DOI:** 10.1136/medethics-2016-103372

**Published:** 2015-10-13

**Authors:** Rebecca C H Brown

**Keywords:** Behaviour Modification, Social Aspects, Health Promotion, Ethics, Public Health Ethics

## Abstract

Financial incentives may provide a way of reducing the burden of chronic diseases by motivating people to adopt healthy behaviours. While it is still uncertain how effective such incentives could be for promoting health, some argue that, even if effective, there are ethical objections that preclude their use. One such argument is made by Michael Sandel, who suggests that monetary transactions can have a corrupting effect on the norms and values that ordinarily regulate exchange and behaviour in previously non-monetised contexts. In this paper, I outline Sandel's corruption argument and consider its validity in the context of health incentives. I distinguish between two forms of corruption that are implied by Sandel's argument: *efficiency corruption* and *value corruption*. While Sandel's thought-provoking discussion provides a valuable contribution to debates about health policies generally and health incentives specifically, I suggest the force of his criticism of health incentives is limited: further empirical evidence and theoretical reasoning are required to support the suggestion that health incentives are an inappropriate tool for promoting health. While I do not find Sandel's corruption argument compelling, this only constitutes a partial defence of health incentives, since other criticisms relating to their use may prove more successful.

## Introduction: chronic disease, behaviour change and health incentives

Worldwide, considerable public resources have been spent on health promotion campaigns to encourage ‘healthy’^i^ lifestyles. Many of these have focused on raising awareness of the links between chronic diseases and behaviours such as smoking, poor diet, lack of physical activity and excessive alcohol consumption. Despite these efforts, many people persist in engaging in such behaviours, and the growing burden of chronic diseases continues to challenge healthcare providers.^1^ Socially deprived groups are more likely to suffer from chronic diseases and their related disadvantages, and so chronic diseases may exacerbate social inequality.

Different approaches to health-related behaviour change have been proposed, including informational and educational strategies, social marketing to shape norms, regulation of industry and subtle environmental interventions that ‘nudge’ people towards healthier behaviours. At least some people engaging in ‘unhealthy’ behaviours would prefer to quit smoking, eat more healthily, exercise more and drink less, and yet struggle to do so. For such people, lack of information may be less obstructive to healthy behaviour change than difficulties inherent in altering socio-culturally engrained habits.^2^

More recently, there has been interest in whether the use of explicit financial incentives can effectively motivate behaviour change.^3–5^ Incentive-based approaches have found support in both social psychological and behavioural economic theories, since they provide immediate, tangible, certain rewards that may help motivate and sustain healthy behaviour adoption.^2^ ^5^ However, evidence about the effectiveness of health incentives is mixed (particularly with regard to habitual behaviours), and ethical objections to their use are sometimes raised.^3^ ^4^ ^6^ ^7^ In this paper, I consider an objection most prominently advocated by Michael Sandel, who argues that permitting transactions in certain contexts—including health-related behaviours—has a corrupting effect on social values that can justify the prohibition of monetary exchange.^8^ ^9^ Throughout this paper, I will use ‘health incentives’ as shorthand to refer to explicit financial or quasi-financial incentives paid to people in exchange for some predetermined change in a health-related behaviour.

Drawing primarily upon Sandel’s book, *What Money Can't Buy: The Moral Limits of* Markets, I first outline Sandel's criticism of the expansion of money markets generally, and of the use of health incentives more specifically. I characterise his concern as including two distinct corruption-based worries, relating to *efficiency corruption* and *value corruption*. Both of these concerns are dependent on empirical evidence, which I suggest is currently insufficient to support Sandel’s argument. Further, I argue that, even if the *value corruption* argument was indicated by the evidence, it is not clear that it provides good reasons to refrain from using health incentives.

## Sandel's corruption argument

In his 2012 book, *What Money Can’t Buy*, Sandel argues that the extension of money markets into areas of life previously not subject to such transactions should, on the whole, be resisted.^8^ ^9^ His claim is, initially at least, appealing. Sandel provides vivid examples of instances where the exchange of money feels, somehow, wrong. Chapters covering topics such as ‘Cash for Sterilization’, ‘Paying Kids for Good Grades’, ‘A Market in Refugees’, ‘Paying to Kill an Endangered Rhino’, ‘Internet Death Pools’ and ‘The Terrorism Futures Market’ illustrate the kinds of exchanges Sandel thinks we should worry about.

Broadly, Sandel's concern is that the explicit involvement of money in certain areas of life not previously governed by monetary exchange can corrupt valuable social norms that typically regulate interactions and exchange in these contexts.^ii^ Sandel diagnoses a historic era of ‘market triumphalism’, now over, but which has left a legacy of money market extension into numerous areas of life:

While it is certainly true that greed played a role in the financial crisis, something bigger is at stake. The most fateful change during the past three decades was not an increase in greed. It was the expansion of markets, and market values, into spheres of life where they don't belong.

What Money Can't Buy, p. 7

Sandel notes two problems arising from this extension of money markets. The first concerns inequality: by increasing the importance of money in distributing a wide range of resources, we reinforce the gap between the rich and poor. I will not directly discuss Sandel's inequality argument here. The second problem Sandel identifies—and which will be the focus of this paper—is the ‘corrosive tendency of markets’. Sandel argues that:

[M]arkets don't only allocate goods; they also express and promote certain attitudes toward the goods being exchanged… when we decide that certain goods may be bought and sold, we decide, at least implicitly, that it is appropriate to treat them as commodities, as instruments of profit and use. But not all goods are properly valued in this way.

What Money Can't Buy, p. 9

The result, Sandel claims, is that by allowing certain things to be allocated according to markets, we commodify and corrupt them. A helpful illustration comes from one of Sandel’s key examples, relating to a study involving Israeli day care centres.

The centers faced a familiar problem: parents sometimes came late to pick up their children. A teacher had to stay with the children until the tardy parents arrived. To solve this problem, the centers imposed a fine for late pickups. What do you suppose happened? Late pickups actually increased.

What Money Can't Buy, p. 64

Sandel explains this surprising effect as resulting from the replacement of one norm with another: pre-fine, parents tried to turn up on time because otherwise they felt guilty for imposing an inconvenience on the teacher. Post-fine, they considered they were simply paying for the additional time the teacher had to stay at work.

Sandel's explanation for the increase in the tendency for parents to arrive late uses motivation crowding theory.^10^ This proposes that incentives substitute an extrinsic motivation for pre-existing intrinsic motivation. In this case, the monetary incentive (to avoid a fine) ‘crowds out’ the motivation previously provided by not wanting to inconvenience the teacher. This may affect both the observed changes in behaviour and unobserved changes to the motivations producing that behaviour.

I think we can distinguish between the two ways in which Sandel thinks motivation crowding can result in corruption. First, *efficiency corruption* results from the rise in undesired behaviour (in this case, the increase in late pick-ups). This is due to motivation crowding creating the opposite from intended effect, and reducing the efficiency of the system. Second, *value corruption* describes the replacement of ‘higher’ norms or values with ‘lower’ ones. In this case, the introduction of a fine provides a new motivation—to avoid the extra payment—which replaces the previous motivation—to avoid inconveniencing teachers. The two effects of *efficiency corruption* and *value corruption* are that, first, the incentive is ineffective at stopping parents from arriving late, and, second, it encourages parents to view teachers as merely instruments providing a service.^9^

### Efficiency corruption and value corruption

The distinction between *efficiency corruption* and *value corruption* is not explicitly drawn by Sandel. They appear, however, to be separate effects that can exist independently of one another, and may provide different reasons for preferring to avoid the use of incentives. For both the *efficiency corruption* and *value corruption* concerns, I am sceptical about the strength and reliability of those reasons. In the case of *value corruption*, this is largely due to empirical observability and the correctness of the explanation for observed effects. I do not object to the claim that, if incentives have the opposite effect on behaviour from that intended, they are probably a poor policy option. However, the likelihood that they will have this effect, and the claim that this results from the corruption of social norms and values, is, I think, overstated by Sandel.

First, motivation crowding theory is contentious. While described in both psychological and economic theories, disagreements persist about whether or not this is a ‘real’ effect, and if the crowding out of motivation is the mechanism responsible for this effect. In the meta-analyses by Cameron and Pierce^11^ and Cameron *et al*,^12^ the authors argue that ‘in general, rewards are not harmful to motivation to perform a task’. This is contrasted with a meta-analysis by Deci, Koestner and Ryan, which showed ‘As predicted, engagement-contingent, completion-contingent, and performance-contingent rewards undermined free-choice intrinsic motivation’.^13^ While I do not have the authority to judge between the disagreements of experienced social scientists, it seems that, though motivation crowding (or a similar effect) may arise in some circumstances, we are not yet in a position to reliably predict what conditions will lead to this and how significant this effect is. A review by Promberger and Marteau sought to explore whether the available evidence indicated that health incentives are likely to crowd out intrinsic motivation, and found that the existing evidence does not support this.^14^

These studies are interested in the behavioural effects of motivation crowding—whether the behaviour of interest increases of decreases with the introduction (and subsequent removal) of incentives. It is still possible that *value corruption* is occurring even if it does not result in an overall reduction in the incentivised behaviour (if, for instance, the extrinsic motivation of the incentive is sufficient to compensate for the reduction in intrinsic motivation). In the case of the day care centres, we must consider whether Sandel’s interpretation of the outcome is correct: whether parents saw the fine as a fee, ceased to feel guilty about arriving late to collect their children and became more likely to think of the teachers in instrumental ways.

Given the information available about the study, it is not possible to confidently answer this question. It is worth noting, however, that the authors of the original paper provide a slightly different explanation for the observed effects:

What this field study teaches us, we believe, is that the introduction of the fine changes the perception of people regarding the environment in which they operate. In particular, we argue that the environment in our study, as in many real-life situations, is defined by an incomplete contract. In the specific situation under examination, the exact consequence of coming late was not specified in the contract between the parents and the day-care centre. For instance, there was no precise set of clauses specifying the consequences of one, two, or several occurrences of a delayed pickup of a child. Parents could form any belief on the matter, as they probably did, and act accordingly.^15^

The fine provides a sort of reassurance to the parents: whereas before, their late arrival might have resulted in the child being abandoned, or their being barred from the centre, or receiving a telling-off or some other unpleasant outcome, now they know they will just have to pay the fine. Also potentially relevant but unmentioned by Sandel is that all of the centres in the study were private, with parents paying fees for their children to attend. The form the fine took was an extra cost added to their monthly bill. The way Sandel presents this example suggests that the money-free relationship between the parents and those caring for their children is corrupted by the introduction of the fine, whereas in fact this relationship was already mediated by money and payment.

*To summarise*: both the *efficiency* and *value* aspects of Sandel's corruption argument have empirical components, which it is not clear are reliably met. Beyond this, however, is the question of whether or not, even if they are met (ie, that norms, values and motivations previously operating are crowded out or replaced by new norms, values and motivations when money is introduced) we should care. This will be key for the *value corruption* argument. In the next section, I will say a little more about value corruption and how convincing this concern is, specifically in the case of health.

## *Value corruption,* health and acting for the ‘Wrong Reasons’

Sandel thinks that norms guiding behaviour—such as not wanting to inconvenience others, or being motivated by altruism—are irreversibly diminished when we allow money markets to spread.^9^ The reduction in prevalence and influence of these norms is to be regretted, aside from their instrumental capacity to drive desirable behaviours. For Sandel, this (along with his concerns about *efficiency corruption*) suggests we should resist the trend for extending money markets into new areas of life, including the use of incentives in health-related contexts, such as undergoing sterilisation, losing weight, bearing children, taking medication and getting vaccinated.^8^ ^9^

Sandel discusses health incentives directly in a section on ‘health bribes’.^9^ The discussion focuses on what I have described as *efficiency corruption*, since Sandel is concerned that paying people to lose weight or quit smoking will be ineffective due to motivation crowding: ‘Paying people to be healthy can backfire, by failing to cultivate the values that sustain good health’.^9^ As discussed, the evidence on motivation crowding, and the effectiveness of health incentives, is indeterminate. It may be the case that, in some circumstances, incentives crowd out intrinsic motivation and reduce desired behaviours, but in others they crowd in that motivation and increase desired behaviours, beyond expectations.^iii^ I will set the *efficiency corruption* concern aside and concentrate instead on the application of the *value corruption* criticism to health incentives.

Sandel thinks health incentives are ‘bribes’ because:

[T]he monetary motive crowds out other, better, motives… Good health is not only about achieving the right cholesterol level and body mass index. It is also about developing the right attitude to our physical well-being and treating our bodies with care and respect. Paying people to take their meds does little to develop such attitudes and may even undermine them.

He goes on to say:

Health bribes trick us into doing something we should be doing anyhow. They induce us to do the right thing for the wrong reason…. But eventually, we should rise above manipulation… If health bribes work, worries about corrupting good attitudes toward health may seem hopelessly high-minded. If cash can cure us of obesity, why cavil about manipulation? One answer is that a proper concern for our physical well-being is a part of self-respect.

What Money Can't Buy, pp. 58–59

Sandel is worried that the explicit involvement of money in some areas of life will encourage those domains to be regulated by monetary, market-based norms and that it will become more common for people to adopt healthy habits in order to boost their bank balance rather than their health. Acting for the ‘wrong reasons’ may be a helpful way of thinking about what I have called the *value corruption* concern. According to Sandel, people ought to adopt healthy lifestyles on the basis of health itself: that to eat well, exercise regularly, not smoke or drink to excess are required for properly respecting (and valuing) one’s physical well-being. Sandel is not explicit, but it seems likely that, on his account, while failure to take care of one's physical health alone may show a lack of self-respect, to subsequently adopt healthy behaviours only because of the introduction of a monetary payment enhances this, perhaps in addition to displaying mercenariness and other similar attitudes.

How confident should we be, however, in making judgements about the ‘right’ and ‘wrong’ reasons for an action and, similarly, in making normative judgements about the desirability of norms regulating health behaviour? If I fail to save a drowning child who I could rescue with relatively little risk or inconvenience to myself, unless someone agrees to pay me £100, I have acted wrongly. My motive for saving the child should be one of human empathy and compassion, not the opportunity for monetary gain. In this case, my failure to save the child in the absence of the incentive makes me deserving of criticism. What does the additional information that I am willing to save the child in exchange for money add? This seems to tell us something about how I value certain outcomes, and my hierarchy of reasons and preferences. In this case I care more about gaining £100 than saving a child’s life. Acting for the wrong reasons, in the context of ‘bribes’, thus consists in both the failure to recognise certain things as valuable to an appropriate extent and the inappropriate valuing of other things.^iv^

There are (at least) two problems with seeking to criticise people on the basis that they act for the wrong reasons. First, we must be able to identify what reasons they are acting on. This is related to the problem of identifying whether and how motivation crowding is responsible for the perverse effect of some incentives. Second, we need some means by which to judge better and worse reasons. In the example above, it may be uncontroversial to suppose that empathy is a better reason to save a life than money, but we do not generally extend this to apply to doctors who work for a salary.

Other authors have discussed related concerns about the involvement of money in certain contexts. Arguments outlined by Margaret Radin, Elizabeth Anderson, Michael Walzer, Deborah Satz and others provide interesting accounts of why some things should not be subjected to monetary exchange. Unfortunately, there is not space to do justice to these discussions here.^v^ Similarly, there is a considerable philosophical literature concerning practical reasons (reasons that guide action). Particularly influential here has been Donald Davidson, who believes that reasons that rationalise a person’s actions typically both *explain* why an agent acted as she did and provide a *justification* for her actions.^23^ Sandel does not provide any analysis of practical reason, nor how health incentives should be conceptualised as providing reasons for action beyond the implicit assumption that they *do* act as reasons, inferred from the fact that they are capable of changing behaviour.

In focusing on Sandel, I only intend my discussion to respond to his account of the way in which money corrupts. This limits the reach of the discussion, since others' related arguments may provide more successful critiques of the use of health incentives. Yet, since Sandel's account of ‘what money can't buy’ has been particularly influential (due, in part, to his public visibility and reach outside academic philosophy) and since the suspicion of health incentives he proposes is reflected in hostility in the media, I think it is worth directly addressing the claims that he makes.^24–27^

Offering incentives to encourage women to breast feed their babies might be seen as an example of the wrong reasons being at work: since breast milk is generally thought to be better for child health, women are advised to breast feed their babies. Those who only breast feed when offered an additional incentive may be criticised for failing to value the health of their child appropriately and caring more about gaining some extra money. A study that interviewed women enrolled on a breastfeeding incentive programme, however, found that many women did not think of themselves as breast feeding ‘for the incentives’.^28^ In fact, they often said the incentive did not affect whether or not they wanted to breast feed. Instead, the women described the incentive scheme as making them feel valued, boosting their mood and facilitating good relationships with support workers. It is not obvious whether or not we should interpret these women as acting for the wrong reasons, since it is not clear how their reasons change and what other factors affected the capacity for their reasons to influence their behaviour.

Even if we have a clear idea of what reasons are motivating people, and how incentives affect this, it will still be difficult to justify ranking those reasons. Some candidate assessment criteria might be that reasons should be internally consistent, self-endorsable, or ones that the agent is able to identify with. But Sandel seems to want something less agent dependent, based around valuing things appropriately. For instance, Sandel's criticism of health bribes rests on the assumption that people ought to adopt certain healthy behaviours anyway, as a part of self-respect and valuing health appropriately.^9^ Yet, if I prefer a sedentary lifestyle to an active one (despite the risks that health promoters claim are associated with my preference) it is not obvious that I am, first, failing to do something I ought to do by not going running and, second, that this shows a lack of self-respect and concern for my health. I simply hold other preferences and values (which may include caring for my health in other ways). There will be those that wish to argue we do have obligations, either to ourselves or others, to try to stay healthy, but this argument is not self-evident, and in claiming that some reasons, norms and values are ‘better’ or ‘higher’ than others, Sandel might be guilty of a degree of chauvinism.

Sandel is generally in favour of non-market norms and against the introduction of money-related norms, yet there are plenty of examples of destructive (non-monetary) social norms in operation. In the case of breast feeding, in some groups this is considered undesirable and taboo, and social norms operate to dissuade women from breast feeding, sometimes with tragic consequences.^29^ ^30^ Anderson also suggests how the norms and values operating in markets may be differently interpreted:

[On one account], free markets are responsible for moral decline, anomie and loneliness, and eat away at their own foundations. [An alternative account] represents capitalism as expanding the scope of cooperation and trust by enabling people to reap the gains from trade worldwide, bridging parochial divisions of nationality, religion, and ethnicity. Capitalism is an engine of cosmopolitanism, cooling socially dangerous passions such as religious fanaticism, and overcoming xenophobia. The impersonality, anonymity, and openness of markets to all comers is favorably contrasted with social orders in which people are tightly constrained by parochial connections and loyalties of family, ethnicity, and neighbourhood.^16^

While Sandel’s arguments about the creep of money markets and market norms into all areas of life sound plausible and potentially alarming, we must be careful to scrutinise his interpretation of the behaviour of people and groups involved in monetary exchange, and not to romanticise the alternative norms and values operating in such contexts.

## Concluding remarks

In this paper I have outlined Sandel’s objection to the general extension of money markets, based around concerns that the introduction of market-based norms and values corrupts pre-existing, non-market norms. I have drawn a distinction between *efficiency corruption* and *value corruption* and, while not denying their plausibility, offered some reasons for being sceptical about the strength and reliability of these criticisms in the context of health incentives. The most valuable contribution of Sandel’s critical discussion of the general extension of money markets, including the use of health incentives, is to provide a useful counterpoint to discussions in economics and health policies that fail to recognise the moral relevance of monetary transactions and consider effectiveness and efficiency as their primary, perhaps the only, concern. I do not, however, think that Sandel's argument as it stands, with the current lack of empirical evidence and theoretical underpinning to support his claims, provides convincing reasons to think that the use of health incentives should be avoided.

## Footnotes

i ‘Healthy’ and ‘unhealthy’ are problematic terms in this context, and would ideally be avoided. However, it is common in health-related literature to use this language as shorthand to refer broadly to behaviours that are generally regarded as decreasing or increasing the risk of suffering from (chronic) disease.

ii Monetary valuations and transactions may implicitly operate in many situations, but for Sandel it is important that the involvement of money is salient.

iii I have not discussed ‘crowding in’, but it is a similar effect to crowding out, except that pre-existing intrinsic motivations for an incentivised behaviour are supported and reinforced when rewards are offered for that behaviour.^10^

iv This fits with others' descriptions of ‘bribes’. For example, Anderson says, “A payment is a bribe when the compensation to the recipient is for a performance that is either not authorized by the relationship or required by the relationship without payment.”^16^

v Very briefly: Radin describes how the market value of property may fail to capture its ‘full’ value (taking into account its personal meaning and significance), since this is incommensurate with a single, monetised valuation.^17^ ^18^ Anderson argues that, since the way we treat things expresses attitudes about how we value those things, subjecting something to monetary exchange expresses the attitude that we value it for its instrumental worth.^19^ ^20^ Anderson is concerned about the consequences of behaviour that expresses inappropriate attitudes towards property, relationships, behaviours and values, by involving them in commodification and monetary exchange. Walzer argues that the ways goods are distributed must be in keeping with their ‘social meaning’, and we change or undermine previous practices of treating certain goods when we introduce monetary exchange as a way of distributing them.^21^ Finally, Satz has an egalitarian-based concern that in ‘noxious’ markets pre-existing power imbalances prevent people from trading as equals.^22^
